# Evolution of Centromeric Retrotransposons in Grasses

**DOI:** 10.1093/gbe/evu096

**Published:** 2014-05-09

**Authors:** Anupma Sharma, Gernot G. Presting

**Affiliations:** Department of Molecular Biosciences and Bioengineering, University of Hawaii, Mānoa

**Keywords:** centromeric retrotransposon, CRM, horizontal transfer, interelement recombination, phylogeny

## Abstract

Centromeric retrotransposons (CRs) constitute a family of plant retroelements, some of which have the ability to target their insertion almost exclusively to the functional centromeres. Our exhaustive analysis of CR family members in four grass genomes revealed not only horizontal transfer (HT) of CR elements between the oryzoid and panicoid grass lineages but also their subsequent recombination with endogenous elements that in some cases created prolific recombinants in foxtail millet and sorghum. HT events are easily identifiable only in cases where host genome divergence significantly predates HT, thus documented HT events likely represent only a fraction of the total. If the more difficult to detect ancient HT events occurred at frequencies similar to those observable in present day grasses, the extant long terminal repeat retrotransposons represent the mosaic products of HT and recombination that are optimized for retrotransposition in their host genomes. This complicates not only phylogenetic analysis but also the establishment of a meaningful retrotransposon nomenclature, which we have nevertheless attempted to implement here. In contrast to the plant-centric naming convention used currently for CR elements, we classify elements primarily based on their phylogenetic relationships regardless of host plant, using the exhaustively studied maize elements assigned to six different subfamilies as a standard. The CR2 subfamily is the most widely distributed of the six CR subfamilies discovered in grass genomes to date and thus the most likely to play a functional role at grass centromeres.

## Introduction

Centromeric retrotransposons (CRs) belong to the gypsy superfamily of long terminal repeat (LTR) retrotransposons and form a clade within the chromoviruses (Gorinsek et al. 2004). The first full-length member of the CR family, the “centromeric retrotransposon of barley,” short “cereba,” was discovered at the centromeres of barley, wheat, and rye (Presting et al. 1998). CR elements are characterized by the presence of the CR motif in their integrase domain, which may target their insertion to specific genomic regions (Gorinsek et al. 2004, 2005; Neumann et al. 2011). Elements belonging to some CR subfamilies are enriched at the centromeres of grass (Miller et al. 1998) and other plant (Weber and Schmidt 2009) species; however, it remains unclear whether centromere targeting has evolved because these insertions are less likely to result in gene disruption or because CR elements actually contribute to centromere function in grasses. Of note here is the recent discovery that CR elements can give rise to tandem repeats (Sharma et al. 2013), which are typical features of most centromeres.

Using the polyprotein region of CR elements, Neumann et al. (2011) showed that these elements were extant at the time of angiosperm/gymnosperm divergence but that the CR motif may have been acquired by elements in the angiosperm lineage only. Four distinct CR subfamilies of maize (CRM) and rice (CRR) have been described as having the following orthologous relationships: CRM1:CRR3, CRM2:CRR2, CRM3:CRR1, and CRM4:CRR4 (Sharma and Presting 2008). CRM1, CRM2, and CRM3 but not CRM4 are enriched in maize centromeres (Schnable et al. 2009). More recently, a fifth CRM clade member, CRM5, was identified (Neumann et al. 2011). CRM5 had previously been described as a 7,892-nt long element with LTRs of length 1,159 and 1,173 bp and was originally named BALTR1 (Lamb et al. 2007). In fluorescent in situ hybridization (FISH) experiments, BALTR1 produced intense hybridization signals in the pericentromeric heterochromatin of *Zea mays* A and the heterochromatin of B chromosomes (Lamb et al. 2007).

We previously showed that interelement recombination between sequence variants created recombinant elements in the CRM1 and CRM4 subfamilies, some of which proliferated after their formation to yield new subgroups (Sharma et al. 2008). Recombination was also documented for Opie elements in maize (Sharma et al. 2008) and several other retrotransposon families in plants (Vicient et al. 2005; Marco and Marin 2008). These recombinations complicate phylogenetic reconstruction, as does horizontal transfer (HT) of retrotransposons, which has been documented for RIRE1 and Route66 elements in the grasses (Roulin et al. 2008, 2009), Rider in tomato (Cheng et al. 2009), and *copia* between *Drosophila* species (Jordan et al. 1999).

We performed an exhaustive characterization of CR elements found in sequenced grass genomes (family Poaceae). Poaceae subfamilies Panicoideae, Pooideae, and Ehrhartoideae (previously known as Oryzoideae) include the majority of the economically important cereal crops of the world. The panicoid lineage includes corn (*Z. mays*), sorghum (*Sorghum bicolor*), job’s tears (*Coix lacryma-jobi*), switchgrass (*Panicum vergatum*), and foxtail millet (*Setaria italica*); the pooid lineage includes purple false brome (*Brachypodium*), oats (*Avena*), brome grasses (*Bromus*), wheat (*Triticum*), barley (*Hordeum*), rye (*Secale*), and other cool season grasses; and the oryzoid lineage includes the cultivated and wild rice (*Oryza* sp.).

Here, we describe a newly discovered sixth CR subfamily of *Z. mays* and reconstruct the phylogenies of all six known maize CR subfamilies and their orthologs in rice, sorghum, and *Brachypodium*. Of particular note is the extraordinary abundance and sequence diversity of CRs in the sorghum genome. We also show that HT of CRs between oryzoid (rice) and panicoid (maize, sorghum, *Setaria*, *Panicum*, and *Coix*) lineages, and recombination between these horizontally acquired and endogenous elements, have played major roles in the evolution and species-specific proliferation of several CR subfamilies.

Because of the sequential nature of element discovery, the current CR nomenclature is fraught with inconsistencies. Different subfamily numbers have been assigned to orthologous subfamilies, and the same name has been assigned to different CRs from two different rice species. For example, *Oryza sativa* CRR1 is homologous to maize CRM3, CRR3 of *O. officinalis* is homologous to CRM1 of maize (Sharma and Presting 2008), and CRR3 of *O. sativa* is homologous to CRM5 of maize (Neumann et al. 2011). In this study, we implement a new nomenclature for the CR elements of grasses that reflects their phylogenetic relationships.

## Materials and Methods

### Discovery of CR Homologs and Copy Number Estimation

BLASTn was used to search the genome assemblies of *Z. mays* (refGen_v2; Schnable et al. 2009), *S. bicolor* (ftp://ftp.jgi-psf.org/pub/compgen/phytozome/v7.0/Sbicolor/assembly/Sbicolor_79.fa.gz, last accessed May 21, 2014; Paterson et al. 2009), *O. sativa* (http://rgp.dna.affrc.go.jp/IRGSP/Build5/build5.html, last accessed May 21, 2014), and *B. distachyon* (ftp://ftp.jgi-psf.org/pub/compgen/phytozome/v7.0/Bdistachyon/assembly/Bdistachyon_114.fa.gz, last accessed May 21, 2014; International Brachypodium Initiative 2010), as well as the nr database of GenBank, for full-length CR elements using CR1-Zm (CRM1), CR2-Zm (CRM2), CR3-Zm (CRM3), CR4-Zm (CRM4) (Sharma and Presting 2008; Sharma et al. 2008), as well as CR5-Zm (CRM5) (Neumann et al. 2011) and CR6-Zm (this study) as queries. Iterative searches were performed to obtain sequence variants of CRs. In addition, we searched for CR1-Zm (CRM1) homologs in the *Se. italica* genome produced by the US Department of Energy, Joint Genome Institute (ftp://ftp.jgi-psf.org/pub/compgen/phytozome/v8.0/Sitalica/assembly/Sitalica_164.fa.gz, last accessed May 21, 2014; Bennetzen et al. 2012).

In general, only full-length copies of maize, sorghum, rice, and *Brachypodium* CRs that were flanked by 5 bp target site duplications (TSDs) containing no more than one mismatch were used in this study. Exceptions to this rule include CR2-Oo and truncated CR1-Oo and CR6-Osj elements, because full-length members of these subfamilies could not be detected in the *O. sativa* ssp. *japonica* genome.

### Choice of Substitution Rates

Assumption of a molecular clock is useful for estimating the divergence time between orthologous molecular sequences. However, uncertainty in the absolute age of the evolutionary event used to calibrate the molecular clock, use of different genes or genomic regions, and different methods to estimate divergence times contribute to the variation in divergence time estimates between species (Gaut 2002). The divergence of the maize, rice, and wheat lineages based on fossil record is estimated at 50–70 Ma (Wolfe et al. 1989). Sequence analysis of duplicated rice genes and expressed sequence tags from related taxa using an average substitution rate of 6.1–6.5 × 10^−^^9^ per synonymous site per year revealed a whole-genome duplication in the common ancestor of grasses approximately 70 Ma (Paterson et al. 2004). To make the divergence times cited in this study as comparable to each other as is practically possible, we assumed maize–rice divergence time of 50 Ma (Wolfe et al. 1989; Gaut and Doebley 1997) and cite divergence times from published studies that either use absolute rates assuming a maize–rice divergence time of 50 Ma or use an average synonymous substitution rate obtained from the grass adh1/2 alleles (6.5 × 10^−^^9^ per site per year) that was estimated by assuming the maize–rice divergence time of 50 Ma (Gaut and Doebley 1997). Where this rule could not be met, we converted the *ks* values derived in other studies to divergence time using the rate estimated from the grass adh1/2. Divergence time estimates for various grass species are listed in supplementary table S1, Supplementary Material online.

Assuming that maize diverged from rice and barley approximately 50 Ma, the synonymous substitution rate (*ks*) of the maize adh1/2 alleles was estimated at average 7.00 ± 1.16 × 10^−^^9^ and 5.99 ± 1.13 × 10^−^^9^ per site per year, respectively (Gaut et al. 1996). The average *ks* value of adh1/2 alleles (6.5 × 10^−^^9^ per site per year; Gaut and Doebley 1997) lies within the range estimated for 11 homeologous maize gene pairs, which varied 2.6-fold ranging from 6.38 ± 0.37 × 10^−^^9^ for the maize pg1 alleles to 16.67 ± 1.69 × 10^−^^9^ for the maize tb1/2 alleles (Swigonová et al. 2004) estimated by assuming that rice diverged from ancestor of sorghum/maize approximately 50 Ma (Wolfe et al. 1989). The average nucleotide substitution rate of intergenic regions is higher than the synonymous substitution in coding regions of genes and justifies the use of a different conversion factor for noncoding versus coding regions. For example, it was proposed that a 2-fold higher substitution rate of 1.3 × 10^−^^8^ mutations per site per year is appropriate to date the insertions of LTR retrotransposons (Ma and Bennetzen 2004), and the nucleotide substitution rate for the tb1 intergenic region of maize was estimated at 2.9–3.3 × 10^−^^8^ (Clark et al. 2005). In this study, the divergence times of different CR subfamilies were estimated using their coding regions using a *ks* value of 6.5 × 10^−^^9^ substitutions per synonymous site per year. Insertion times of individual full-length retrotransposons into the genome were estimated as previously described (SanMiguel et al. 1998) using the sequence divergence of the two LTRs and the rate of 3.3 × 10^−^^8^ substitutions per site per year derived for the tb1 intergenic region of maize (Clark et al. 2005).

### Phylogenetic Reconstruction

MUSCLE (http://www.ebi.ac.uk/Tools/msa/muscle/, last accessed May 21, 2014; Edgar 2004) was used to generate a multiple sequence alignment (MSA) of 2,450 nt from the polyprotein region spanning the RT domain to the polypurine tract of 410 CR elements (2 from *B. vulgaris*, 6 from *Arabidopsis* and all others from grasses; supplementary file S1, Supplementary Material online). The MSA was edited manually, using BioEdit (Hall 1999), to remove single nucleotide in-dels and then used to generate neighbor joining phylogenetic tree using the Tamura 3-parameter model implemented in MEGA5 (Tamura et al. 2011). Bootstrapping was conducted with 1,000 replicates.

### Divergence Time Estimation

The MSA used to generate the phylogenetic tree was also used to estimate the codon-based evolutionary divergence based on the number of synonymous substitutions per synonymous site (*ks*) between CR subfamilies/subgroups and between CR clades using the Nei–Gojobori (Jukes–Cantor) method implemented in MEGA5 (Tamura et al. 2011). The ambiguities or gaps were deleted in pairwise manner, and the bootstrap method was used to estimate the variance/standard error using 500 replications. First, CR elements were grouped by subfamily/subgroup to compute the number of synonymous substitutions per synonymous site (*ks*) over sequence pairs between groups. This analysis involved 410 nucleotide sequences from 57 groups/CR subfamilies (including eight groups from dicots *Arabidopsis*/*B. vulgaris*) and a total of 611 positions in the final data set. Next, the CR elements were grouped by clades (CR1 through CR6 and cereba) to compute the codon-based evolutionary divergence or the number of synonymous substitutions per synonymous site (*ks*) over sequence pairs between groups. This analysis involved 404 sequences from seven grass CR clades (only two *Arabidopsis* CRA3 members were included as outgroup) and a total of 615 positions in the final data set. The *ks* values thus obtained were converted to divergence time using the synonymous substitution rate of the maize adh1/2 alleles, that is, *r* = 6.5 × 10^−^^9^ substitutions per synonymous site per year (Gaut and Doebley 1997). The *ks* values and the corresponding divergence times are listed in supplementary file S2, Supplementary Material online. The box plot showing distribution of divergence time of oryzoid/panicoid CR subfamily pairs was plotted using R (R Core Team 2012).

### Estimation of CR Element Insertion Times

The insertion time of CR elements was calculated based on nucleotide difference between the 5′- and 3′-LTRs of LTR retrotransposons, an approach pioneered by SanMiguel et al. (1998). The number of substitutions per nucleotide site (*k*) between the 5′- and 3′-LTRs of full-length elements (with recognizable TSD) was calculated, using the Kimura 2-parameter model implemented in MEGA5 (Tamura et al. 2011) and converted to insertion time using the nucleotide substitution rate for the tb1 intergenic region of maize, that is, 3.3 × 10^−^^8^ (Clark et al. 2005). The insertion times estimates using the nucleotide substitution rate for the tb1 intergenic region of maize are 5-fold lower than those using the synonymous substitution rate of the grass adh1/2 allele, which was used to estimate insertion time of retrotransposons in the pioneering (SanMiguel et al. 1998) and several other studies (Sharma et al. 2008; Ma and Bennetzen 2004).

### Sequence Similarity Plots

The MSA of the recombinant CR and related CR subfamilies was generated using MUSCLE (Edgar 2004). The similarity of the recombinant sequence/subfamily to other related CRs was plotted using SimPlot (Lole et al. 1999) using a sliding window of 200 nt and step size of 20. The percent identity between pairs of CRs was calculated by the NCBI BLAST2seq program.

### Domain Structure of CR2-Sb Subfamily Members

An MSA of CR2-Sb elements was generated using MUSCLE (Edgar 2004) and then subdivided into 13 subalignments based on LTR and polyprotein domains. CR2-Sb sequences that always formed separate groups were arbitrarily called the “A” and “B” parental types, and each subdomain of the remaining recombinant sequences was scored as either A or B based on its grouping with the parent types A or B.

### Identification and Phylogenetic Analysis of CR Homologs in Barley

The barley whole-genome shotgun assembly at http://mips.helmholtz-muenchen.de/plant/barley/index.jsp (last accessed May 21, 2014) (assembly3_WGSMorex_with 2,670,738 sequences; 1,868,648,155 total letters) was searched using BLASTn and maize CRs. The top hit, which was always to the polyprotein region, was extracted and aligned to maize CRs. The MSA of these sequences with the consensus sequences of the six maize CR subfamilies and Beetle1 and Beetle2 elements was generated using MEGA5 (Tamura et al. 2011) and manually edited using BIOEDIT (Hall 1999) to remove gaps, and flushed to generate a 2,447-nt long alignment of spanning the RT domain to the polypurine tract. Maximum parsimony and neighbor joining trees were generated in MEGA5 (Tamura et al. 2011) using 1,000 bootstrap replicates. The Tamura 3-parameter model was used to compute evolutionary distance for the neighbor joining tree.

### d*S* and d*N* Ratio

The ratio of d*S* (number of synonymous substitutions per synonymous site) to d*N* (number of nonsynonymous substitutions per nonsynonymous site) was calculated from d*S* and d*N* values estimated using Nei–Gojobori method (Jukes–Cantor) implemented in MEGA.

## Results

### A Revised Nomenclature for CRs

**Fig. 1.—:**
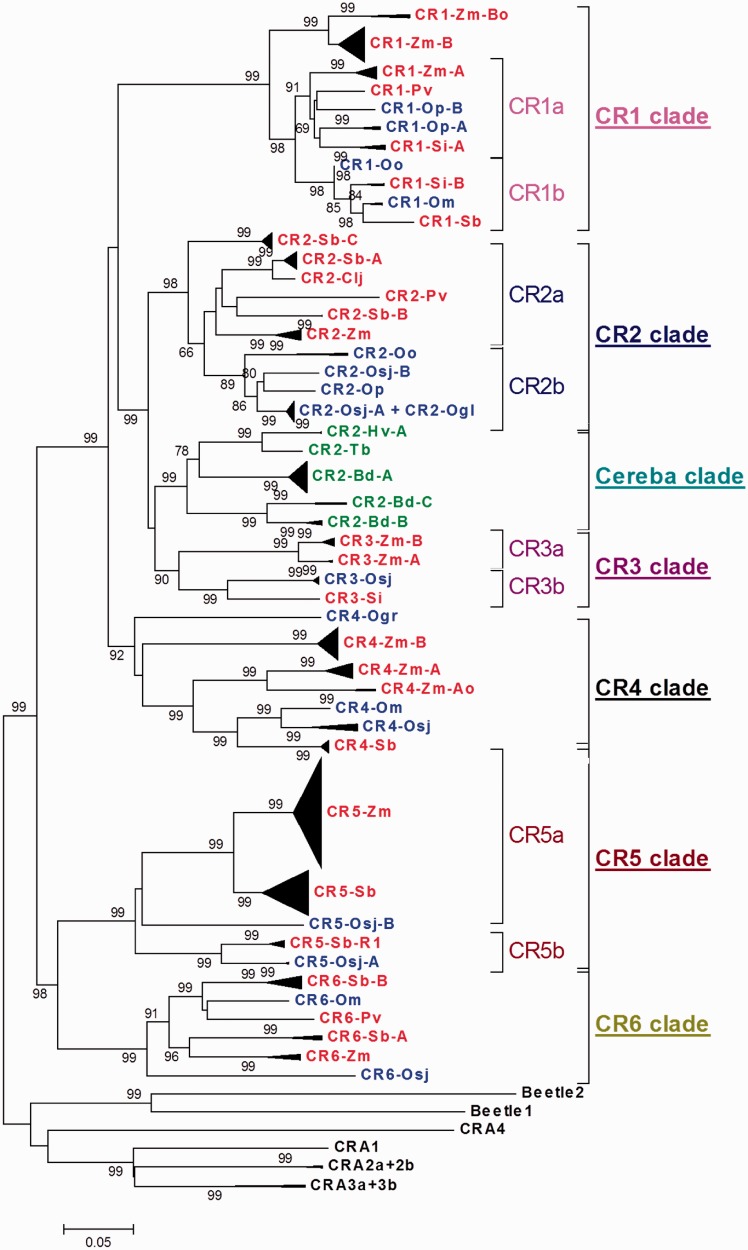
Neighbor-joining phylogenetic tree showing seven CR clades belonging to six CR subfamilies. The tree is based on nucleotide sequence spanning the polyprotein-coding region from the RT domain through polypurine tract. CRs belonging to the same subfamily are collapsed and shown as black triangles at terminal nodes. The height of triangle indicates the total number of full-length elements. Bootstrap percentages obtained after 1,000 replicates are indicated on nodes. All CRs included in the tree except CR2-Oo and the two truncated CR1-Oo and CR6-Osj elements are flanked by intact TSDs.

We implemented a tripartite nomenclature for CRs of grasses that includes 1) the CR subfamily name based on the maize naming convention (e.g., CR1 for elements related to CRM1), 2) the host species name (abbreviated genus and species/subspecies initials, e.g., Zm for *Z. mays*, Osj and Osi to indicate japonica, and indica subspecies of *O. sativa*, etc.), and 3) subgroup name (e.g., R1 for recombinant 1). In this system, the CR subfamily name is assigned based on sequence homology and phylogenetic relationship to the maize CR subfamilies (fig. 1), using the polyprotein coding region spanning the reverse transcriptase domain to the polypurine tract. Furthermore, a subgroup name is assigned to phylogenetically distinct sequence variants within a CR subfamily, either alphabetically (A, B, C, etc.) in the order of their discovery or alphanumerically (e.g., R1, R2, and R3) if the subgroups are determined to have originated via recombination between two extant sequence variants. Thus, the notation “CR1-Zm-A” denotes sequence variant A of the *Z. mays* (Zm) CR1 subfamily, which was previously called CRM1A (Sharma et al. 2008).

### Identification of New Maize CR Family Member CR6-Zm and CR Homologs in Other Grasses

CR6-Zm is a previously uncharacterized pericentromeric member of the CR family. We detected five full-length CR6-Zm elements in RefGen_v2 (supplementary table S2, Supplementary Material online) that inserted into the maize genome between 0.850 ± 0.124 and 1.193 ± 0.158 Ma. (The two LTRs of each full-length element differ by an estimated 0.056 ± 0.008 to 0.079 ± 0.010 nucleotide substitutions per site [*k*].) Each of the five elements is flanked by 5-nt long TSDs and none of them encode an intact full-length ORF. The polyprotein-coding region of CR6-Zm elements contains numerous stop codons and deletions. The most structurally intact copy of CR6-Zm (CR6-Zm_3), at RefGen_v2 chr1 coordinates 126,489,540–126,481,843, is 7,698-nt long with LTRs of 1,162 nt. Neither the full-length nor partial copies of CR6-Zm or CR5-Zm are enriched in the maize centromeres as defined by chromatin immunoprecipitation with the centromere-specific histone H3 variant CENH3 (data not shown). Thus, the total complement of maize CR elements includes six distinct lineages that are named CR1-Zm through CR6-Zm.

We searched for homologs of the five previously described (CR1-Zm through CR5-Zm, formerly CRM1 through CRM5), and the newly discovered CR6-Zm, maize elements in the publicly available sequenced genomes of *S. bicolor*, *O. sativa* ssp. *japonica*, and *Brachypodium distachyon*, as well as in the nonredundant (nr) database of GenBank. Homologs of the six maize CR subfamilies are present in both oryzoid and panicoid lineages. Supplementary table S3, Supplementary Material online, lists all CR elements identified in this study and those described in previous studies (including sequence variants) with their historical names and references.

### Phylogenetic Reconstruction Yields Six Distinct CR Subfamilies

A bootstrapped neighbor joining tree was constructed from 402 full-length autonomous CR elements identified in maize, rice, sorghum, *Brachypodium*, *Setaria*, *Panicum*, *Coix*, and wild rice species using approximately 2,450 nt of the polyprotein-coding region from the RT domain to the polypurine tract (hereafter referred to as RT-PPT) and rooted on CR elements of the dicots *Beta vulgaris* and *Arabidopsis thalina* (fig. 1, sequences available in supplementary file S1, Supplementary Material online). Recombinant elements identified in this or previous studies (e.g., CR1-Zm-[R1-R5], CR4-Zm-R1, CR2-Sb-[R1-R12], and CR1-Si-[R1,R1del,R2]) were excluded from the phylogenetic analysis if representatives of both parents could be identified in the respective genome, but included if one of the parental elements was determined to have been eliminated from the host genome (e.g., CR5-Zm, CR5-Sb-R1, and CR6-Sb-A). When all known recombinants derived from two parents shared the same polyprotein sequence, only one recombinant subgroup was included to represent the set (e.g., CR5-Sb-R1 also represents CR5-Sb-[R2-R4]).

Phylogenetic reconstruction grouped the CR elements into seven easily recognizable clades that formed around CR1-Zm through CR6-Zm and cereba (fig. 1). Clades CR1 through CR6 contain members from both panicoid and oryzoid lineages (fig. 1 and supplementary table S3, Supplementary Material online), whereas the cereba clade contains only pooid lineage members. Because cereba clade members are most similar (as measured by synonymous distance, see supplementary file S2, Supplementary Material online) to members of the CR2 clade, we have classified these elements as CR2s. Therefore, of the six CR subfamilies, the CR2 subfamily has the largest host range among the grass genomes examined here.

### Evidence for HT of CR Elements between the Panicoid and Oryzoid Lineages

Several phylogenetic incongruities supported by pairwise sequence comparisons and synonymous distance estimates provide evidence for HT of CR elements both within and between members of the oryzoid and panicoid lineages. A general description of each clade is provided below, and instances of likely HT are highlighted.

#### The CR1 Clade: Evidence for Ancient and Recent HT between the Oryzoid and Panicoid Lineages

The maize CR1-Zm-B elements (and the related B_o_ variants) are unique to maize and are located at the base of the CR1 clade, which bifurcates into two additional subclades, “CR1a” and “CR1b,” both of which contain members from the panicoid and oryzoid lineages (fig. 1). Within the CR1 clade, *Z. mays* CR1-Zm-B and *O. officinalis* CR1-Oo subfamilies exhibit the highest estimated divergence time (46.075 ± 6.589 Ma, see supplementary file S2, Supplementary Material online), which is close to the estimated divergence time of the panicoid:oryzoid lineages (∼50 Ma). However, the grouping of panicoid with oryzoid lineage members in subclades CR1a and CR1b is unexpected, because the two grass lineages diverged much earlier than the *Oryza* species approximately 15 Ma (Ammiraju et al. 2008; Bertoni 2008).

Within the CR1a clade, the *O. punctata* CR1 (CR1-Op-A/B) groups with the panicoid lineage members. The divergence time between oryzpoid/panicoid CR1a clade pairs are low (average 19.780 Ma and ranging from 14.984 ± 1.854 Ma for CR1-Op-A/CR1-Si-A to 24.589 ± 2.669 for CR1-Op-A/CR1-Sb; supplementary file S2, Supplementary Material online) and comparable to that among some oryzoid pairs (e.g., CR1-Op-A and CR1-Op-B of CR1a subclade diverged 21.145 ± 2.353 Ma; CR1-Op-A and CR1-Op-B of CR1a subclade diverged from CR1-Oo and CR1-Om of subclade CR1b between 19.502 ± 2.325 and 26.804 ± 4.382 Ma; supplementary file S2, Supplementary Material online). The unexpectedly low sequence divergence between these oryzoid–panicoid CR1a clade members (see supplementary fig. S1*a* and *b* and file S2, Supplementary Material online) is likely not due to an exceptionally low synonymous substitution rate, since the divergence time estimates of CR1-Zm-B and CR1-Oo are consistent with the oryzoid–panicoid genome divergence time. Instead, we suspect HT of a CR1a clade member approximately 30 Ma that allowed it to sweep through the panicoid and/or oryzoid lineages via a complex pattern of frequent HT and recombination events that are difficult to reconstruct, in part because CR1 clade members appear to have been lost from some genomes or remain to be identified in as yet incompletely sequenced panicoid species (e.g., homologs of CR1-Zm-B elements have not been identified in any other sequenced genome).

**Fig. 2.—:**
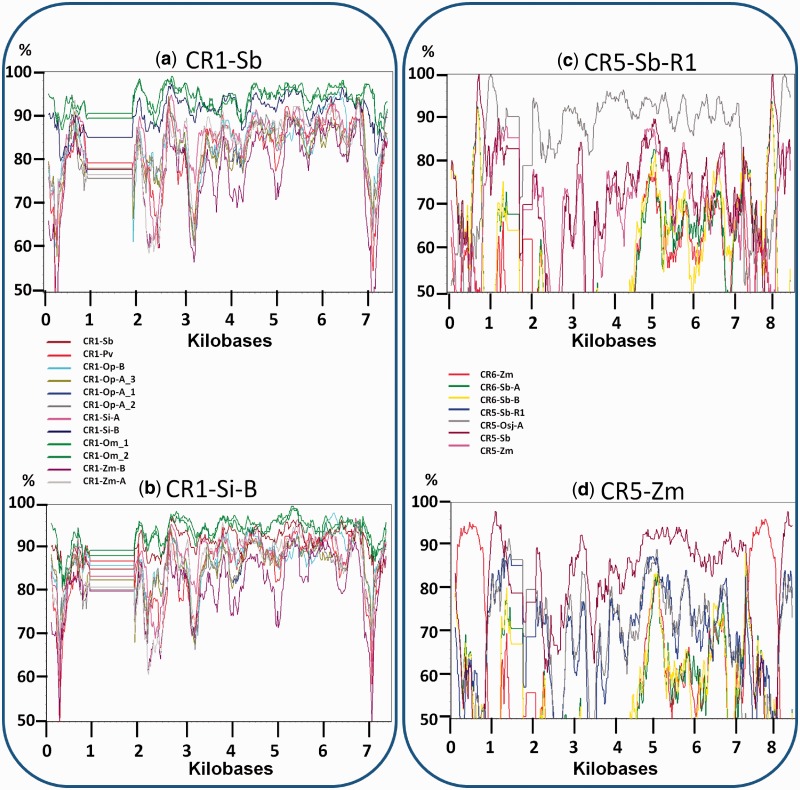
SimPlots of CR subfamilies derived by HT and/or interelement recombination. The percent similarity of a given CR subfamily relative to other related CR subfamilies is plotted in moving windows of 200 nt with step size 20. Straight lines, such as in the UTR regions, indicate gapped region in multiple sequence alignment due to higher divergence of the UTR between CR subfamilies. *X* axis indicates distance along sequence alignment and is marked in increments of 1,000 nt each starting from position 0; *Y* axis indicates percent similarity and is marked in increments of 10% starting from 50 to 100. (*a*) *Sorghum bicolor* CR1 clade member “CR1-Sb” shares high sequence similarity to *O. minuta* CR1 clade member “CR1-Om” (dark green and light green) throughout its length. (*b*) *Setaria italica* CR1 clade member “CR1-Si-B” shares high sequence similarity to O. minuta CR1 clade member “CR1-Om” (dark green and light green) throughout its length. (*c*) *Sorghum bicolor* CR5 clade member “CR5-Sb-R1” has high sequence similarity to *O. sativa* CR5 clade member “CR5-Osj-A” (gray) except at the 5′-end of LTR, which is homologous to another *S. bicolor* CR5 clade member “CR5-Sb” (purple) and to *S. bicolor* CR6 clade members “CR6-Sb-A/B” (yellow and green). (*d*) *Zea mays* CR5 clade member “CR5-Zm” has high sequence similarity to *S. bicolor* CR5 clade member “CR5-Sb” (purple) except in part of LTR at the beginning, which is similar to *Z. mays* CR6 clade member “CR6-Zm.”.

Divergence of oryzoid–panicoid CR1 pairs from subclade CR1b is even more recent (average 4.767 Ma, and ranging from 2.880 ± 0.792 for CR1-Om/CR1-Sb to 7.245 ± 1.931 for CR1-Oo/CR1-Si-B). Within the CR1b clade, *S. bicolor* CR1 (CR1-Sb) and one of the *Se. italica* CR1s (CR1-Si-B) group most closely with *O. minuta* CR1 (CR1-Om). Sequence similarity plots of full-length CR1 clade members support the phylogenetic grouping of *S. bicolor* CR1-Sb, *Se. italica* CR1-Si-B, and *O. minuta* CR1-Om, as CR1-Sb and CR1-Si-B have higher sequence identity to CR1-Om over their entire length (>92% in LTR and >94% identity in the coding region of each pair) than to other panicoid CR1 elements (fig. 2*a* and *b*). The divergence times of CR1-Sb and CR1-Si-B from CR1-Om are estimated at approximately 2.880 ± 0.792 and 4.125 ± 1.005 Ma, respectively (supplementary file S2, Supplementary Material online). These data suggest recent HT of CR1b clade members between panicoid and oryzoid lineage members at approximately 4 Ma between *Setaria* and *Oryza* and at approximately 3 Ma between sorghum and *Oryza*.

The divergence time between CR1-Zm-A and CR1-Zm-B was estimated between 13 (using *r* = 1.67 × 10^−^^8^ [Swigonová et al. 2004]) and 34 Ma (using *r* = 6.5 × 10^−^^9^ [Gaut and Doebley 1997]) based on *ks* value of 0.4343 obtained for the ORF of the most closely related CR1-Zm-A and CR1-Zm-B elements, and thus we had previously proposed that the two CR1 subgroups of maize may have diverged as a consequence of the divergence of the two maize progenitors approximately 11.9 Ma (Swigonová et al. 2004; Sharma et al. 2008). However, divergence times relative to known CR1 clade members show that CR1-Zm-A has the highest synonymous distance to CR1-Zm-B (among all CR1 clade members) and is more similar to *Se. italica* CR1-Si-A and CR1-Si-B, *O. minuta* CR1-Om, and *O. punctata* CR1-Op (supplementary file S2, Supplementary Material online). The fact that CR1-Zm-B is basal to all other known CR1 clade members (fig. 1) and unique to maize suggests that CR1-Zm-B represents an ancient CR1 clade member that diverged early and was retained only in maize, or was acquired by maize via HT from a distantly related unknown source.

#### The CR2 Clade: HT within the Oryzoid and Panicoid Lineages

The sorghum CR2-Sb-C elements are located at the base of the CR2 clade, which bifurcates into two additional subclades, “CR2a” and “CR2b” (fig. 1). Clade CR2a contains only panicoid lineage members, whereas clade CR2b is composed exclusively of oryzoid lineage members (fig. 1). The estimated divergence times for oryzoid–panicoid CR2 pairs range from 32.748 ± 4.275 to 51.135 ± 4.919 Ma (supplementary file S2, Supplementary Material online), which is in line with the estimated divergence time of the panicoid:oryzoid lineages. Similarly, average divergence times of CR2 clade members within the panicoid (ranging from 5.854 ± 1.110 to 45.191 ± 5.599 Ma) and the oryzoid (ranging from 1.373 ± 0.479 to 19.615 ± 2.255 Ma) lineages agree with the divergence time estimates based on gene phylogenies and reflect the relatively recent speciation within the genus *Oryza* estimated to have occurred within the past 10–15 Myr (Ammiraju et al. 2008; Bertoni 2008; Zou et al. 2008). Thus, the available sequence data do not support HT of CR2 elements between the panicoid and oryzoid lineages.

However, there is evidence of HT of CR2 subfamily members within the same lineage. The *O. sativa* ssp. *japonica* genome contains two CR2 clade members, CR2-Osj-A and CR2-Osj-B (supplementary table S3, Supplementary Material online, and fig. 1)—the latter is represented in this genome by a single full-length copy that inserted an estimated 0.996 ± 0.147 Ma (*k = *0.066 ± 0.010, supplementary fig. S2, Supplementary Material online). Sequence similarity plots illustrate that CR2-Osj-B is very similar to the *O. officinalis* CR2 (CR2-Oo) in the 3′-end of the LTR, part of the UTR, and the gag and protease encoding region of the polyprotein but more similar to *O. sativa* CR2-Osj-A in the remainder of the sequence (supplementary fig. S1*c*, Supplementary Material online). Specifically, CR2-Osj-B shares approximately 87% sequence identity with CR2-Oo in the 1,886 nt region of the polyprotein region spanning the gag and protease domains but only 71% sequence identity with CR2-Osj-A over 1,797 nt in the same region. Thus, CR2-Osj-B appears to be a CR2-Oo-like element in which part of the polyprotein region (including RT domain through polypurine tract) has been replaced with that from *O. sativa* CR2-Osj-A. Because CR2-Osj-B is a recombinant element, its divergence to CR2-Oo was calculated using the gag (20.967 ± 2.449 Ma) and protease (16.392 ± 5.105 Ma) domains, and its divergence to CR2-Osj-A was calculated at 12.330 ± 1.528 Ma using the RT-PPT region (supplementary table S4 and file S2, Supplementary Material online). Both estimates for the CR2-Osj-B/CR2-Oo divergence time are higher than that for the *O. sativa* (AA genome) and *O. officinalis* lineages (CC genome) estimated at average from 5.3 to 11.2 Ma (Ammiraju et al. 2008), which is not unusual as different genes are under different selective constraints, and because gene trees are often older than the species tree (Nichols 2001).

It remains unclear how *O. sativa* ssp. *japonica* acquired two CR2 variants, one of which exhibits higher sequence similarity to *O. officinalis* CR2 than the other. One possibility is that the rice lineage contained two CR2 variants, both of which are represented (one as recombinant) in *O. sativa.* Alternatively, an early HT event may have led to the transfer of the CR2-Osj-B progenitor from a wild rice species (related to *O. officinalis*) to an *O. sativa japonica* progenitor, followed by recombination between the horizontally acquired CR2-Osj-B progenitor and the endogenous CR2-Osj-A element of *O. sativa*. In this context, it is noteworthy that recent HT of RIRE1 between *O*. *australiensis* and seven other reproductively isolated *Oryza* species has been documented (Roulin et al. 2008).

Similarly, the divergence of *S. bicolor* CR2-Sb-A and *C. lacryma-jobi* CR2-Clj is estimated at 5.854 ± 1.110 Ma (supplementary file S2, Supplementary Material online), whereas phylogenetic reconstruction based on full-length sequences of the phytoene synthase 1 (PSY1) gene suggests that the Coix lineage splits from the common ancestor before the divergence of maize and sorghum (Fu et al. 2010), which is estimated at approximately 11.9 Ma (Swigonová et al. 2004). Thus, we conclude that there is some evidence of potential HT of CR2 elements within the panicoid (*Coix* and sorghum) and the oryzoid (*O*. *officinalis* and *O*. *sativa*) lineages.

#### The Cereba Clade: Pooid Lineage Members Only

The cereba clade is closely related to the CR2/CR3 clades of the oryzoids and panicoids but comprised of members from the pooid lineage only, that is, *Brachypodium*, *Triticum*, and *Hordeum* (fig. 1). The average synonymous distance of cereba clade members to CR2 clade members is lower than to the CR3 clade members (supplementary file S1, Supplementary Material online), indicating that the cereba clade represents the CR2 subfamily in the pooid lineage.

*Brachypodium distachyon* contains three distinct cereba variants that we assigned to different subfamilies: CR2-Bd-A, CR2-Bd-B, and CR2-Bd-C. CR2-Bd-A diverged from CR2-Bd-B and CR2-Bd-C about 55.698 ± 5.725 and 57.895 ± 6.284 Ma respectively, whereas CR2-Bd-B and CR2-Bd-C diverged more recently at approximately 15.510 ± 2.058 Ma (supplementary file S2, Supplementary Material online). CR2-Bd-B and CR2-Bd-C share approximately 86% sequence identity. The *B. distachyon* genome (2 *n* = 10) seems to have a homoploid origin via ancestral interspecific hybridization (Wolny et al. 2011; Catalán et al. 2012). Similarly, interspecific hybridization between *B. distachyon* (2 *n* = 10) and *B. stacei* (2 *n* = 20) formed the allotetraploid *B. hybridum* (2 *n* = 30) (Catalán et al. 2012). Thus, the different cereba lineages of *B. distachyon* may have originated via interspecific hybridization between different *Brachypodium* species at different times. Although all three cereba clade members of *Brachypodium* are centromeric (data not shown), only CR2-Bd-A subfamily members have recently increased in number at *Brachypodium* centromeres; the few CR2-Bd-B and CR2-Bd-C subfamily members that do exist are old (supplementary fig. S2, Supplementary Material online).

Barley cereba elements (GenBank accession number AY040832), which we named CR2-Hv-A for consistency, are phylogenetically closer to wheat CR2-Tb than to the CR2 elements of *Brachypodium*. CR2-Hv-A and CR2-Tb share 84–85% sequence identity and diverged an estimated 18.921 ± 2.175 Ma. The *Triticum*/*Hordeum* divergence time was estimated at approximately 6.2–10.9 Ma (Ramakrishna et al. 2002; Chalupska et al. 2008).

All noncereba CR elements appear to have been lost in *Brachypodium*, but at least some of them have been retained in other pooid lineage members. For example, phylogenetic trees constructed from CR polyproteins identified in barley and the maize CR reference elements show the presence of CR4, CR5, and CR6 clade members in addition to three distinct cereba clade members in barley (supplementary fig. S3, Supplementary Material online) but no CR1 or CR3 clade members. The two new cereba clade members have approximately 86% sequence identity to the original cereba elements (accession numbers AY040832.1) and 83% sequence identity to each other in the RT-PPT region.

#### The CR3 Clade: The *O. sativa* and *S*e*. italica* CR3 Homologs Are more Similar to Each Other than Either Is to Maize

The CR3 clade contains very few elements and bifurcates into subclade “CR3a,” which contains the two *Z. mays* CR3 variants CR3-Zm-A and CR3-Zm-B, and subclade “CR3b,” which contains the *O. sativa* and *Se. italica* CR3 (fig. 1). CR3-Zm-A (more similar to CentA [Ananiev et al. 1998]) and CR3-Zm-B diverged approximately 9.484 ± 1.444 Ma (supplementary file S2, Supplementary Material online), close to the divergence time (∼11.9 Ma) of the two parents that eventually formed current day maize by allotetraploidization and were likely reunited in the maize genome during that event. Recombinant CR3-Zm elements were not detected and may not have formed due to the low number of CR3-Zm elements in the maize genome. A CR3 homolog was not detected in the sequenced sorghum genome analyzed here.

Sequence similarity plots support the phylogenetic grouping of CR3 homologs from *O. sativa* ssp. *japonica* (CR3-Osj) and *Se. italica* (CR3-Si) (supplementary fig. S1*d*, Supplementary Material online). CR3-Si shares an overall 83% sequence identity with CR3-Osj in a 5,230-nt long region spanning the complete polyprotein and 472 nt of the 5′-UTR, as well as 78% and 80% sequence identity in a 436-nt and a 196-nt long segments of the LTR, respectively. In contrast, CR3-Si shares only 77% sequence identity with maize CR3 (CR3-Zm) in a 5,104-nt long region spanning the polyprotein and approximately 79% sequence identity in a 333-nt long region of LTR.

The *Z. mays* CR3-Zm-A and CR3-Zm-B lineages diverged from *Se. italica* CR3 an estimated 72.222 ± 7.647 and 72.775 ± 7.471 Ma, respectively, and from *O. sativa* CR3 at 80.381 ± 8.832 and 80.729 ± 8.856 Ma (supplementary file S2, Supplementary Material online), somewhat earlier than the estimated divergence time (∼50 Ma) of the oryzoid and panicoid lineages (Wolfe et al. 1989; Gaut and Doebley 1997). The *O. sativa* and the *Se. italica* CR3 diverged around 46.298 ± 4.521 Ma, which is consistent with the estimated divergence time of oryzoid and panicoid lineages. The abnormally high divergence time estimate of CR3-Zm may be due to acquisition of CR3-Zm by maize from an unknown distant source, possibly via HT. Alternatively, CR3-Zm may be an ancient paralog of *O. sativa* and *Se. italica* CR3.

#### The CR4 Clade: The *O. sativa* and *S. bicolor* CR4 Homologs Are more Similar to Each Other than Either Is to Maize

The *O. granulata* CR4 (CR4-Ogr) and one subgroup of the *Z. mays* CR4 subfamily (CR4-Zm-B) are located near the base of the CR4 clade (fig. 1). As in the case of CR1-Zm-B, no CR4-Zm-B homolog has been identified in the nonmaize plant genomes analyzed thus far. *Sorghum bicolor* CR4 (CR4-Sb) groups more closely with the *O. sativa* and *O. minuta* CR4s (CR4-Osj and CR4-Om) than with either maize CR4 (CR4-Zm-A/B).

Sequence similarity plots support the phylogenetic grouping of the *S. bicolor* CR4 (CR4-Sb) with *O. sativa* CR4 (CR4-Osj) (supplementary fig. S1*e*, Supplementary Material online), which share 82% sequence identity in a 4,882-nt region spanning the complete polyprotein and 227 nt of the 5′-UTR, as well as 78% identity in a 919-nt LTR region. On the other hand, CR4-Sb shares only 77% sequence identity with CR4-Zm-A over a 4,279-nt region spanning the complete polyprotein and 77% and 74% identity in two regions of the LTR that are 416 and 263 nt long, respectively.

The estimated divergence time for CR4s of *S. bicolor* and *Z. mays* (∼62.349 ± 6.012 to 78.791 ± 8.830 Ma) is much larger than the estimated divergence time between CR4s of *S. bicolor* and *O. sativa* or *O. minuta* (∼28.981 ± 3.060 to 34.859 ± 3.783 Ma) (supplementary file S2, Supplementary Material online). The higher sequence similarity of *S. bicolor* CR4-Sb to rice CR4-Osj versus maize CR4 could be due to an ancient HT event.

The estimated divergence time of CR4-Zm-A and CR4-Zm-B (∼83.012 ± 9.528 Ma) is much earlier than that of the two maize progenitor genomes (∼11.9 Ma) that were united in the most recent allotetraploidization event, disproving an earlier proposition that these two CR4 variants arose after divergence of their host genomes (Sharma et al. 2008).

#### The CR5 Clade: HT of CR5 from Rice to Sorghum Was Followed by Recombination with the Endogenous Sorghum CR5/CR6 Elements

**Fig. 3.—:**
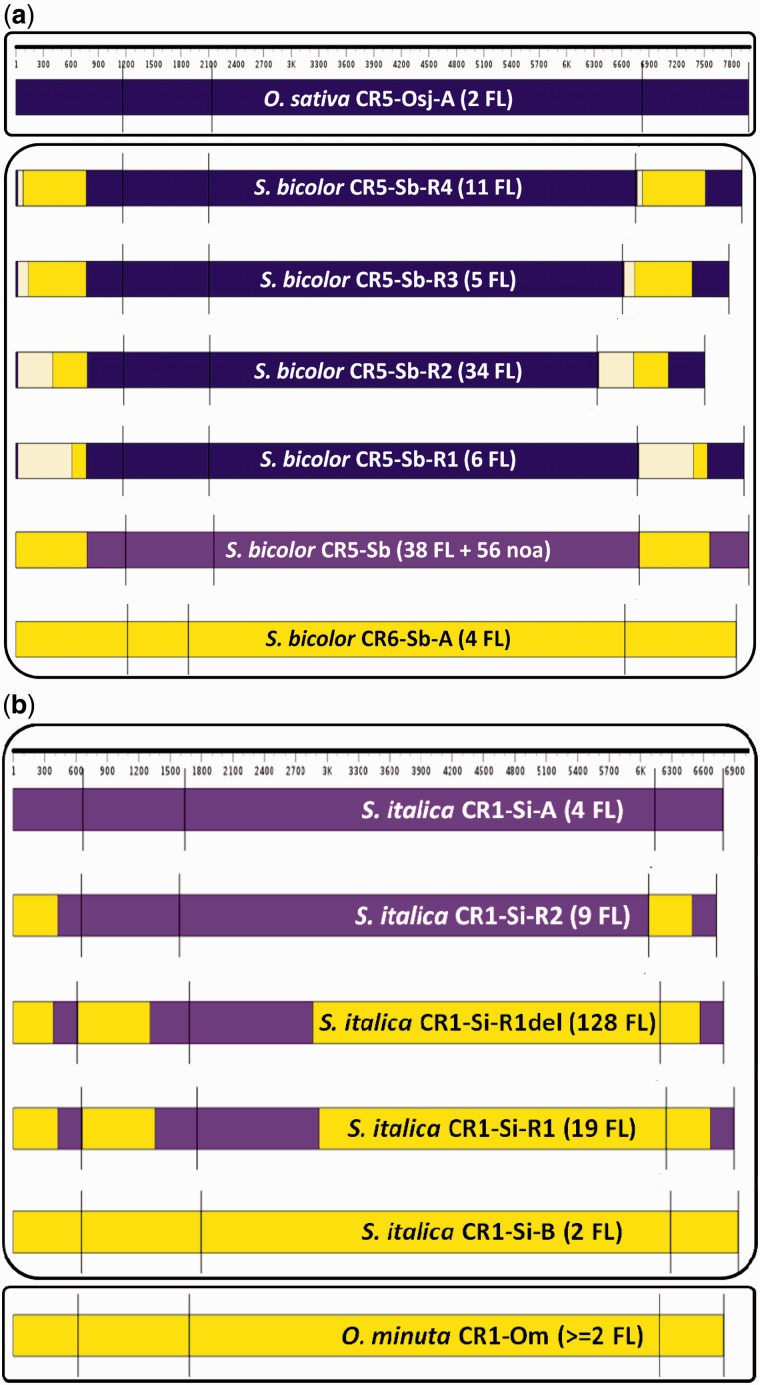
Sequence relationship of recombinant sorghum CR5 and *Setaria* CR1 elements to their respective parents. The CR elements are drawn to scale, and vertical bars mark the ends of the 5′-LTR, 5′-UTR, polypurine tract preceeding the 3′-LTR and the 3′-LTR. (*a*) Four recombinant subgroups of sorghum CR5-Sb-[R1-R4] (i.e., R1–R4) are derived from sorghum CR5-Sb/CR6-Sb and rice CR5-Osj-A subfamilies. CR5-Osj-A was transferred to the sorghum genome via HT, where it recombined with CR5-Sb element to form CR5-Sb-R1 recombinants 1–4. CR5-Sb itself is thought to be a recombinant of CR6-Sb-A and a CR5-Sb progenitor that could not be detected. The oldest CR5-Sb-[R1-R4] member inserted 0.900 ± 0.116 Ma, and older members of parent CR5-Osj-A subfamily appear to have been lost from the sequenced *Oryza sativa* ssp. *japonica* genome. Only two young members of CR5-Osj-A family less than 0.026 ± 0.018 Ma could be detected in the current rice genome assembly. (*b*) Three recombinant subgroups of *Setaria italica* CR1-Si (R1, R1del, and R2) are derived from *Se. italica* CR1-Si-A and an *O. minuta* CR1-Om-like element (CR1-Si-B) that was transferred to the *Se. italica* genome via HT.

Sorghum CR5 clade members form two distinct phylogenetic groups based on their polyproteins. CR5-Sb groups with maize CR5 (CR5-Zm) to form clade “CR5a,” whereas the remaining CR5 clade members of sorghum, CR5-Sb-[R1-R4], group with *O. sativa* CR5 (CR5-Osj-A) in clade “CR5b” and are represented by CR5-Sb-R1 in figure 1. (Because the four recombinant subgroups CR-Sb-R1, -R2, -R3, and -R4 differ primarily in a region of their LTRs [see fig. 3 for a schematic], the latter three were not included in the phylogenetic tree that was generated using polyprotein sequences.) Divergence of CR5-Sb and CR5-Sb-R1 is estimated at 75.416 ± 9.401 Ma. In contrast, divergence of sorghum CR5-Sb-R1 and *O. sativa* CR5-Osj-A is much more recent (26.168 ± 3.070 Ma) and similar to that of *S. bicolor* CR5-Sb and maize CR5-Zm (26.034 ± 2.840 Ma) (supplementary file S2, Supplementary Material online).

Sequence similarity plots of the entire element illustrate that CR5-Sb-R1 is a recombinant that is most similar to rice CR5-Osj-A throughout, except in a part of the LTR, which is similar to that of sorghum CR5-Sb and CR6-Sb-A/B elements (fig. 2*c*). Specifically, sorghum CR5-Sb-R1 shares 89% sequence identity with rice CR5-Osj-A over 6,008 nt that span 407 nt of LTR and the complete UTR and polyprotein but only 79% and 74% sequence identity with maize CR5-Zm over 3,560 nt (polyprotein) and 2,254 nt (LTR and UTR). Thus, CR5-Sb-R1 and related recombinant subgroups (R2, R3, and R4—see fig. 3*a* for schematic) apparently arose via recombination between a horizontally acquired CR5 element from rice (CR-Osj-A like element) and the endogenous sorghum CR5 elements (CR5-Sb of subclade CR5a) and/or sorghum CR6 clade members. These results illustrate that novel and prolific CR subfamilies can originate by the concerted action of HT and recombination with endogenous elements in a new host.

*Oryza sativa* also has two distinct CR5 clade members, CR5-Osj-A and CR5-Osj-B, that share 82% sequence identity in the polyprotein region. Although the 3′-end of the CR5-Osj-B LTRs is similar to CR5-Osj-A (∼70% identity in roughly 600 nt at the 3′-end), its 5′-end is similar to that of the rice CR6 clade member CR6-Osj (78% identity over 400 nt near the 5′-end). CR5-Osj-B thus appears to be an ancient recombinant derived from a CR5-Osj-A-like element and a CR6-Osj-like element of rice (see supplementary fig. S1*f*, Supplementary Material online). We detected only two full-length copies of CR5-Osj-A and a single full-length copy of CR5-Osj-B, all of which inserted less than 0.026 ± 0.018 Ma (*k* = 0.000 ± 0.000 or 0.002 ± 0.001) (supplementary fig. S2, Supplementary Material online).

#### The CR6 Clade: Evidence of Ancient HT Followed by Recombination

In general, autonomous full-length CR6 elements are relatively rare in the sequenced genomes and tend to be old and degenerate, making analysis of this group especially challenging. The youngest full-length CR6 clade members inserted about 0.195 ± 0.052 Ma (*k = *0.013 ± 0.003) (supplementary fig. S2, Supplementary Material online). Interestingly, a region of the CR6 subfamily LTR has been incorporated independently into CR5 via recombination in at least three grass genomes analyzed here, that is, rice (detailed above), sorghum, and corn (detailed below).

The *O. sativa* CR6 element CR6-Osj (for which no full-length members were detected) is located at the base of the CR6 clade. Remarkably, the *O. minuta* CR6 element (CR6-Om) grouped with the panicoid CR6 elements and most closely with sorghum CR6-Sb-B elements. Phylogenetic grouping of CR6-Om and CR6-Sb-B polyproteins is supported both by sequence similarity plots (supplementary fig. S1*g–i*, Supplementary Material online) and divergence time estimates. CR6-Om exhibits 89% sequence identity to CR6-Sb-B, but only 85% to CR6-Sb-A, 87% to CR6-Pv, and 83% identity to CR6-Zm (supplementary fig. S1*i*, Supplementary Material online) in the 2,440 nt RT-PPT region used for both phylogenetic reconstruction and divergence time estimation. CR6-Om divergence from CR6-Sb-B, CR6-Sb-A, CR6-Pv, and CR6-Zm is estimated at 33.616 ± 3.753, 59.346 ± 6.442, 41.638 ± 4.098, and 65.353 ± 7.749 Ma, respectively, suggesting ancient HT of a CR6 member from *O*. *minuta* to sorghum.

However, the fact that the two sorghum CR6 clade members do share similar LTR, UTR, and gag sequences and differ only in the approximately 2,900 nt long polyprotein region that includes the RT, RNaseH, and integrase domains (supplementary fig. S1*g–j*, Supplementary Material online) suggests that one or both of the sorghum elements have undergone recombination. We detected 11 CR6-Sb-B group members that inserted between 0.195 ± 0.052 and 1.232 ± 0.165 Ma (*k* = 0.013 ± 0.003 and 0.081 ± 0.011) and 4 CR6-Sb-A elements that inserted between 0.671 ± 0.096 and 1.032 ± 0.122 Ma (*k = *0.044 ± 0.006 and 0.068 ± 0.008).

### Most Horizontally Transferred CR Subfamilies Are under Purifying Selection

Members of a CR clade generally show higher sequence similarity within the same plant lineage than across different lineages (e.g., oryzoid and panicoid). Nevertheless, we identified four cases where CR elements were more similar between the panicoid and oryzoid lineages than expected from the divergence time of these lineages (i.e., pairs CR1-Sb/CR1-Om, CR1-Si-B/CR1-Om, CR4-Sb/CR4-Osj, and CR5-Sb-R[1-4]/CR5-Osj-A), likely due to HT. We estimated the d*N*/d*S* ratio, which is an indicator of selective pressure on protein-coding genes, for each of these four CR pairs (supplementary table S5, Supplementary Material online).

The d*N*/d*S* ratio is less than one for all four CR pairs, indicating purifying selection or selection against deleterious nonsynonymous substitutions. However, the d*N*/d*S* ratio of the CR1-Sb/CR1-Om (d*N*/d*S* = 0.738) pair is much higher than those of the other three pairs (d*N*/d*S* ranging from 0.128 to 0.341), which more closely match the d*N*/d*S* ratios estimated for seven pairs of genes between rice and maize (d*N*/d*S* ranging from 0.023–0.280), rice and sorghum (d*N*/d*S* = 0.035–0.247) and sorghum and maize (d*N*/d*S* = 0.017–0.226) (Roulin et al. 2009), and for two pairs of genes between *Setaria* and rice (d*N*/d*S* = 0.125 and 0.139) (Diao et al. 2006). The relatively high d*N*/d*S* ratios of the CR1-Sb/CR1-Om pair seem to be a consequence of inactivity of the CR1-Sb subfamily, that is, only one full-length copy (disrupted by a large insertion) and a truncated copy of CR1-Sb were detected in the sorghum genome assembly. Thus, CR1-Sb seems not to have been fixed in the sorghum genome and may be on the verge of extinction. Based on the low d*N*/d*S* ratios of the other three CR pairs, similar to genes, we conclude that CRs from these three pairs are either active or were recently active in their respective host genomes. This conclusion is also supported by the exceptionally high estimated insertion time for the sole CR1-Sb copy (1.757 ± 0.255 Ma, *k = *0.116 ± 0.017) compared with those elements belonging to the other four CR pairs examined here (insertion times ranging from 0.026 ± 0.018 to 0.688 ± 0.147 Ma) (supplementary fig. S2, Supplementary Material online).

### Recombination Has Generated Novel and Prolific Retrotransposon Subgroups

Previously we documented proliferation of at least five recombinants derived from the A and B variants of the CR1-Zm subfamily and one recombinant derived from A and B variants of the CR4-Zm subfamily (Sharma et al. 2008). Here, we identified several new cases of recombination mediated shuffling of retrotransposon segments involving 1) two variants of the same CR subfamily (e.g., CR2-Sb-A and CR2-Sb-B in sorghum), 2) members of different CR subfamilies (e.g., a CR5 progenitor and CR6 in maize, a CR5-Sb progenitor and CR6-Sb-A/B in sorghum, and CR5- and CR6-like elements in rice), or 3) a horizontally acquired retrotransposon and its corresponding endogenous retrotransposon (e.g., an *O. sativa* CR5-Osj-A-like element and sorghum CR5-Sb-R1 progenitor, and an *O. minuta* CR1-Om-like element and *S. italic*a CR1-Si-A element). In many cases, recombination resulted in the formation of active elements that proliferated in the respective host genomes.

#### Recombination between a Horizontally Acquired CR1 and the Endogenous CR1 Subfamily Resulted in Proliferation of at least Two Recombinants in Se. italica

*Setaria italica* contains at least two distinct CR1 elements—an endogenous CR1-Si-A subfamily and the CR1-Si-B subfamily that was acquired by HT from *O. minuta*. Recombination between the A and B variants of CR1-Si resulted in proliferation of recombinant subgroups CR1-Si-R1, CR1-Si-R1del, and CR1-Si-R2 (fig. 3*b*). We identified 4 full-length copies of CR1-Si-A (*k* = 0.035 ± 0.008 to 0.051 ± 0.009 or 0.526 ± 0.118 to 0.770 ± 0.140 Ma), 2 of CR1-Si-B (*k* = 0.045 ± 0.010 to 0.049 ± 0.010 or 0.688 ± 0.147 to 0.743 ± 0.156 Ma), 19 of CR1-Si-R1 (*k = *0.006 ± 0.003 to 0.058 ± 0.011 or 0.095 ± 0.046 to 0.875 ± 0.172 Ma), 128 of CR1-Si-R1del (a deletion derivative of CR1-Si-R1 with *k = *0.000 ± 0.000 to 0.046 ± 0.010 or 0.000 ± 0.000 to 0.699 ± 0.147 Ma), and 9 of CR1-Si-R2 (*k = *0.029 ± 0.007 to 0.049 ± 0.009 or 0.443 ± 0.103 to 0.745 ± 0.141 Ma) (supplementary fig. S2, Supplementary Material online), each flanked by intact TSDs.

#### Recombination between Two Variants of the CR2-Sb-A and CR2-Sb-B Resulted in Formation and Proliferation of at least 12 Different Recombinant Subgroups in S. bicolor

*Sorghum bicolor* contains three distinct CR2 homologs, that is, CR2-Sb-A, CR2-Sb-B, and CR2-Sb-C (supplementary table S3, Supplementary Material online). Recombinations between CR2-Sb-A and CR2-Sb-B have generated at least 12 different recombinant subgroups. We identified 15 copies of CR2-Sb-A (*k = *0.011 ± 0.003 to 0.037 ± 0.006 or 0.165 ± 0.048 to 0.563 ± 0.098 Ma), 14 copies of CR2-Sb-B (*k = *0.007 ± 0.003 to 0.018 ± 0.004 or 0.099 ± 0.044 to 0.276 ± 0.059 Ma), and 110 copies from the 12 recombinant subgroups, that is, CR2-Sb-[R1-R12]. These 110 recombinants are currently active and have inserted into the sorghum genome in the past 0.662 ± 0.102 Ma (*k = *0.044 ± 0.007) (supplementary fig. S2, Supplementary Material online). The domain structure of CR2-Sb-[R1-R12] recombinants is illustrated in supplementary figure S4, Supplementary Material online. Unlike CR2-Sb-A, the majority of CR2-Sb-B subfamily members are nonautonomous, that is, their RT and integrase domains are either absent or truncated. Moreover, CR2-Sb-A itself appears to be an ancient recombinant between a CR2-Sb-B- and CR2-Sb-C-like element (supplementary fig. S1*k*, Supplementary Material online).

#### Recombination between a Maize CR5 Progenitor and CR6 Element Resulted in the Proliferation of a Recombinant CR5-Zm Subfamily

The present-day maize CR5 subfamily arose by recombination between an element of a CR5 subfamily (i.e., no longer present as complete elements in the inbred B73 genome) and a member of the extant CR6 subfamily. Two truncated LTRs of the ancestral CR5 subfamily were identified in the maize genome, and these remnant LTRs have homology to rice CR5-Osj-A LTRs (data not shown). Figure 2*d* illustrates the recombinant nature of CR5_Zm. We identified a total of 93 full-length copies of CR5-Zm flanked by TSDs. These CR5-Zm elements integrated into the maize genome between 0.037 ± 0.024 and 1.103 ± 0.148 Ma (*k = *0.002 ± 0.002 to 0.073 ± 0.010) (supplementary fig. S2, Supplementary Material online).

#### Recombination between the Endogenous Sorghum CR5 and CR6 Subfamilies Resulted in the Proliferation of Recombinant CR5-Sb Elements in S. bicolor

CR5 elements of sorghum are grouped into two types based on their polyprotein, that is, CR5-Sb and CR5-Sb-[R1toR4]. CR5-Sb LTRs are similar to those of CR6-Sb-(A/B) and thus seem to have originated by recombination between an extinct endogenous sorghum CR5 element and CR6-Sb-A/B (fig. 3*a* and supplementary fig. S1*l*, Supplementary Material online). The sorghum genome contains 38 copies of CR5-Sb that inserted between 0.107 ± 0.041 and 1.279 ± 0.159 Ma (*k = *0.007 ± 0.003 to 0.084 ± 0.011) and another 56 copies of noaCR5-Sb elements (nonautonomous derivatives of CR5-Sb) that inserted between 0.090 ± 0.037 and 1.280 ± 0.141 Ma (*k = *0.006 ± 0.002 to 0.084 ± 0.009) (supplementary fig. S2, Supplementary Material online).

#### Recombination between a Horizontally Acquired CR5 and the Endogenous CR5 or CR6 Subfamily Resulted in Proliferation of Four Recombinant Subgroups in S. bicolor

The CR5-Sb-R1, -R2, -R3, and -R4 subgroups of sorghum originated by recombination between a horizontally acquired rice CR5-Osj-A-like element with an endogenous CR5/CR6 clade member of sorghum (details discussed above, also see figs. 2*c* and 3*a*). We identified 6 copies of CR5-Sb-R1 (*k = *0.011 ± 0.003 to 0.054 ± 0.008 or 0.173 ± 0.045 to 0.818 ± 0.121 Ma), 34 copies of CR5-Sb-R2 (*k = *0.005 ± 0.002 to 0.056 ± 0.008, or 0.078 ± 0.031 to 0.852 ± 0.0117 Ma), 5 copies of CR5-Sb-R3 (*k = *0.013 ± 0.004 to 0.044 ± 0.007 or 0.200 ± 0.059 to 0.669 ± 0.101 Ma), and 11 copies of CR5-Sb-R4 (*k = *0.012 ± 0.003 to 0.059 ± 0.008 or 0.187 ± 0.047 to 0.900 ± 0.116 Ma) (supplementary fig. S2, Supplementary Material online).

### Older CR Copies Are Purged from the Genome

Retrotransposons suffer deletions and mutations over time, and intact old elements are very rare (Ma et al. 2004; Baucom et al. 2009). We determined the copy number and minimum, maximum, and average insertion time of each CR subfamily included in the phylogenetic tree, as well as the recombinant CR2-Sb-[R1-R12] and CR5-Sb-[R1-R4] elements (supplementary fig. S2, Supplementary Material online). The oldest intact (i.e., free of long insertions) full-length CR element identified in this study (CR1-Zm-B element at refGen_v2 chr5 coordinates 106554945–106561781) inserted 1.407 ± 0.229 Ma (*k = *0.093 ± 0.015).

The ability to date retrotransposon insertion times (SanMiguel et al. 1998) provides an independent mechanism to verify our classification of parental and recombinant elements. In most cases, recombinant elements were confirmed to be younger than their parents. However, some recombinant CR2-Sb-[R1-R12] elements are older (0.662 ± 0.102 Ma, *k = *0.044 ± 0.007 for the oldest full-length member) than both their parents, that is, CR2-Sb-A (0.563 ± 0.098, *k = *0.037 ± 0.006 for the oldest full-length member) and CR2-Sb-B (0.276 ± 0.059 Ma, *k = *0.018 ± 0.004 for the oldest full-length member), possibly because old copies of CR2-Sb-A and CR2-Sb-B were eliminated from the *S. bicolor* genome. We believe that our assignment of CR2-Sb-A and CR2-Sb-B subfamilies as parents is correct because CR2-Sb-A and CR2-Sb-B differ in sequence throughout their lengths (supplementary fig. S1*k*, Supplementary Material online), whereas the recombinants have a mosaic arrangement of CR2-Sb-A- and CR2-Sb-B-derived sequence (supplementary fig. S4, Supplementary Material online).

## Discussion

### Evidence for HT of CRs between Oryzoid and Panicoid Lineages

**Fig. 4.—:**
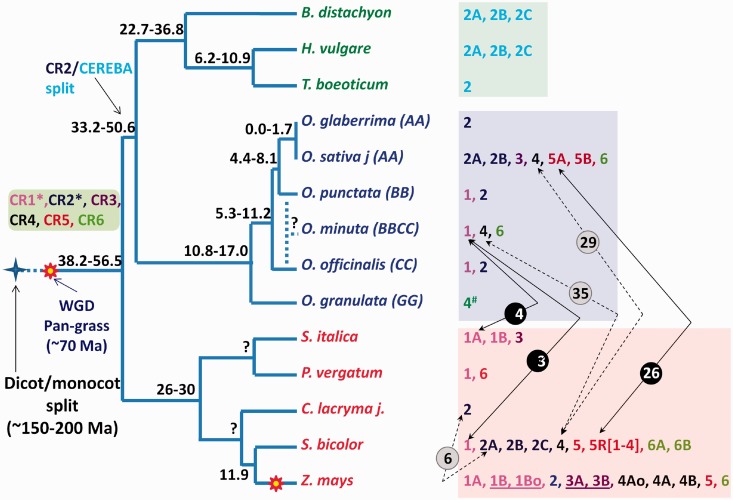
Model of CR subfamily evolution in grasses. A model of CR subfamily evolution is presented with respect to the speciation tree of three grass lineages—the panicoids (species names in red), oryzoids (species names in blue), and pooids (species name in green). Species divergence times on the nodes are from publications referenced in supplementary table S1, Supplementary Material online. The known repertoire of CR subfamilies from completely and partially sequenced grass species is shown to the right of each terminal node. Solid (high confidence) and dotted (low confidence) lines with double-sided arrows connect CR pairs likely involved in HT. The circled numbers over each connecting line indicate the divergence time estimate for the CR subfamilies/subgroups connected by each line. Divergence of CR pairs -CR1-Sb/CR1-Om, CR1-Si-B/CR1-Om, CR2-Sb-A/CR2-Clj, CR5-Sb-R[1-4] (recombinants)/CR5-Osj-A, CR4-Sb/CR4-Osj, and CR4-Sb/CR4-Om is estimated at 2.880 ± 0.792, 4.125 ± 1.005, 5.854 ± 1.110, 26.168 ± 3.070, 28.981 ± 3.060, and 34.859 ± 3.783 Ma, respectively. The underlined CR pairs are those with divergence time (9.573 ± 1.621 Ma for CR1-Zm-B and CR1-Zm-Bo and 9.484 ± 1.444 Ma for and CR3-Zm-A and CR3-Zm-B) less than the estimated time of divergence of the two maize progenitors from each other and from sorghum approximately 11.9 Ma and thus likely diverged with and later reunited with the two maize progenitors in the present-day maize genome. Low-confidence HT events not shown here include the ancient sweep of a CR1 clade member through oryzoid and panicoid lineage members, ancient HT within the CR6 clade, and the within oryzoid HT of a CR2 clade member (see text for details). Our data suggest that CR3, CR4, CR5, and CR6 lineages split into at least two variants between 80.729 ± 8.856 and 95.039 ± 12.833 Ma, followed by the divergence of CR1 and CR2 subfamilies into the extant variants approximately 50 Ma. Indirect evidence (*), based on estimated divergence time to CR3, CR4, and CR5 subfamilies, suggests that CR1 and CR2 subfamilies existed prior to the pan-grass whole-genome duplication (see text for details).

We have identified at least three clear cases of HT of CRs between the oryzoid and panicoid lineages (fig. 4). Genomic DNA that has been transferred between reproductively isolated species is marked by much higher sequence conservation than the remainder of the genome. An average sequence identity of 85% (ranging from 81.9% to 89.1%) was estimated for the coding regions of seven pairs of orthologous sorghum and rice genes (Roulin et al. 2009), and the average sequence identity between the exons of three pairs of conserved genes from *Se. italica* and rice was estimated to be 83.3% (ranging from 74% to 88%) (Diao et al. 2006). The two most recent HT events involving CR between oryzoid and panicoid lineages are supported by the high sequence identities (i.e., > 94% in ∼4,993-nt polyprotein region of CR1-Sb/CR1-Om and >95% in ∼4,642-nt polyprotein region of CR1-Si-B/CR1-Om) that far exceed those between highly conserved orthologous gene pairs from the respective host genomes. The third clear case of HT is the acquisition of CR5-Osj-A-like polyprotein into sorghum recombinant subgroups CR5-Sb-R[1-4]. Although the sequence identity of CR5-Osj-A polyprotein to the CR5-Sb-R[1-4] elements is similar to that expected for a highly conserved gene, and only slightly higher (∼90% in coding sequence) than those of other putative cases of HT described in this study, additional evidence supports this third HT event. First, CR5-Sb appears to be the true ortholog to the only maize CR5 clade member, CR5-Zm. Second, the divergence estimate for CR5-Sb-R[1-4] and rice CR5-Osj is comparable to that of CR5-Sb and CR5-Zm, even though sorghum diverged much earlier from rice than maize. Third, the divergence time of CR5-Sb-R1/CR5-Osj-A pair is significantly lower (extreme outlier in box plot, supplementary fig. S5, Supplementary Material online) than those of all other oryzoid/panicoid CR5 subfamily pairs (ranging from 69.035 ± 7.166 for CR5-Sb/CR5-Osj-B to 81.210 ± 9.152 for CR5-Zm/CR5-Osj-A). The two most recent and clear cases of HT of CR1 clade members do not appear as extreme outliers in the box plot (supplementary fig. S5, Supplementary Material online) because a sweep of CR1a clade members across the oryzoid and panicoid lineages approximately 30 Ma resulted in low divergence time estimates for the majority of CR1 clade members.

Geographical proximity of donor and host is a prerequisite for HT, and our inability to find HT events involving members of the Pooid clade may be due to geographic isolation, as these plants generally prefer a more temperate habitat. Rice, corn, and sorghum, on the other hand, likely shared habitats during the time period when HT is estimated to have occurred. Human cultivation of sorghum and rice crop species began only in the last approximately 10,000 years (Zohary and Hopf 2000; Murphy 2007) and almost certainly did not play a role in the HT events described here and estimated, based on the insertion time of the oldest elements in each subfamily involved, to have occurred more than 281,000 ± 86,000 years ago (*k* values range from 0.002 ± 0.002 to 0.019 ± 0.006 for CR1-Om, 0.116 ± 0.017 for the sole CR1-Sb, 0.045 ± 0.010 to 0.049 ± 0.010 for CR1-Si-B, and 0.005 ± 0.002 to 0.059 ± 0.008 for the recombinant CR5-Sb-[R1-R4] elements).

### Mechanism of HT

Viruses represent important vehicles of horizontal gene transfer in both animals and bacteria (Becker 2000; Lindell et al. 2004; Liu et al. 2010, 2011). LTR retrotransposons resemble retroviruses in both structure and replication mechanism, and thus, a mechanism similar to viral infection might be involved in HT of retrotransposons between plants. Alternatively, HT could be mediated by pollen. Grasses are wind pollinated, and there are several reports of viable offspring from sorghum–rice crosses (Deming et al. 1985; Anon 1989; Srivastava and Yoshida 1990), although the genetic make-up of these progeny is unknown. Oat and wheat pollinated with maize can yield viable embryos (Riera-Lizarazu et al. 1996; Kynast et al. 2001). Although the resulting oat–maize addition (OMA) lines eventually lose most or all maize chromosomes, it is conceivable that retrotransposons jump from one genome to the other while both sets of chromosomes share a nucleus. Although the production of OMA lines requires in vitro embryo rescue, viable seed might be produced in similar wide crosses if unreduced megaspores were involved. Furthermore, the likelihood that these distant pollinations resulted in viable seed is expected to have been higher in the past, when the two genomes would have been more closely related. In fact, maize itself is the product of an allotetraploidization event between two ancestral genomes estimated to have diverged about 11.9 Ma (Swigonová et al. 2004).

### Direction of HT between the Panicoid and Oryzoid Lineage

Sorghum and rice predominantly self-pollinate but do outcross at rates of more than 0.1% for *O. sativa* (Endo et al. 2009), an average of 10.20% for *O. rufipogon* (Phan et al. 2012), 18% for cultivated sorghum, and between 5% and 40% for sorghum landraces (Barnaud et al. 2008). Although sorghum is more closely related to maize than rice, sorghum CR1 is more similar to CR1s of *O. minuta* and *O. officinalis*, one of the two sorghum CR5 variants is more similar to *O. sativa* than to maize CR5, and sorghum CR4 elements are more similar to their counterparts in rice than to those in maize, all of which suggests that the direction of HT was from rice to sorghum. Similarly, the presence of two CR1 clade members in *Se. italica*, one of which is more similar to *O. minuta*, suggests that HT in this case also originated from rice.

### New Insights into Retrotransposon Evolution

Several cases of HT involving retrotransposons (Jordan et al. 1999; Roulin et al. 2008, 2009; Cheng et al. 2009) as well as interelement recombination between CR subfamily alleles resulting in formation and proliferation of novel recombinant retrotransposons have been documented earlier (Sharma et al. 2008), but our comprehensive analysis of the CR family of elements across a number of host genomes has revealed the first example of horizontally transferred elements recombining with endogenous elements to create prolific new recombinants. The findings presented in this study illustrate that both HT and interelement recombination are frequent and that retrotransposons can use these mechanisms in concert to increase their copy numbers.

The sum of newly discovered and previously described recombinant retrotransposons suggests that recombination is more frequent between variants of the same CR subfamily (e.g., those of CR1-Zm, CR4-Zm, and CR2-Sb subfamilies) than between subfamilies (e.g., maize CR5-Zm is derived from an extinct CR5-Zm precursor and CR6-Zm, sorghum CR5-Sb is derived from an extinct CR5-Sb precursor and CR6-Sb-A/B, and *O. sativa* CR5-Osj-B is derived from CR5-Osj-A and CR6-Osj-like elements). It is remarkable that recombination between CR5 and CR6 elements occurred independently in the genomes of maize (fig. 2*d*), sorghum (fig. 2*c* and supplementary fig. S1*l*, Supplementary Material online), and rice (supplementary fig. S1*f*, Supplementary Material online), as evidenced by different recombination breakpoints in the respective recombinant elements, which may indicate that these related CRs are cotranscribed and copackaged, allowing for template switching (see review by Buzdin [2004]). Alternatively, closely related CRs may target similar genomic regions, thereby increasing the likelihood of nested insertions and formation of novel recombinants by illegitimate recombination as proposed previously (Sharma et al. 2008).

### HT, Recombination, and Phylogenetic Classification of CR Subfamilies

HT and recombination of retroelements present special challenges to phylogenetic classification of retrotransposons in general and CR elements in particular. In phylogenetic reconstruction, recombinant elements will group differently depending on which region of the element is used. Removal of older (parental) CR elements from the genome within several million years of their insertion further complicates determination of the evolutionary history of retrotransposons. This is illustrated by CR1-Zm-A (formerly CRM1A), which was previously identified as the parental element that generated CR1-Zm recombinants 1–5 (Sharma et al. 2008). CR1-Zm-A shares high sequence identity (96% over 414 nt and 98% over 924nt) with CR1-Zm-B in a region spanning the 3′-end of LTR, the UTR, and the 5′-end of polyprotein (supplementary fig. S1*m*, Supplementary Material online), illustrating that CR1-Zm-A itself is a recombinant. The original sequence of the replaced CR1-Zm-A segment is unknown as the CR1-Zm-A progenitor appears to have been lost in maize. We thus propose that initial phylogenetic classification of retrotransposons into subfamilies be based on the longest easily alignable sequence fragments, which in the case of CR elements spans from the RT domain to the PPT, and to include related elements regardless of plant host, followed by detailed sequence analysis using similarity plots of these elements, as described here.

### Caveats of Estimating CR Subfamily Divergence Times

Tracing the evolutionary history of CRs relies on accurate determination of their insertion and divergence times, which is a function of substitution rates. However, substitution rates differ between loci and between organisms. For example, the synonymous substitution rates varied from 0% to 2.5% among a set of 24 randomly chosen orthologous genes from the *indica* and *japonica* subspecies of *O. sativa* (Ma et al. 2004). Similarly, the synonymous substitution rate varied nearly 2.6-fold for orthologs of 11 maize genes (Swigonová et al. 2004). LTR retrotransposons are subject to slightly higher mutation rate compared with genes due to the relatively high error rate of the reverse transcriptase and the higher replication frequency compared with the host genome. The highly variable (upto five orders of magnitude) synonymous substitution rates observed previously among 49 different species of RNA viruses was attributed to differences in their replication frequency, as the rates of replication error is roughly constant over different RNA viruses (Hanada et al. 2004). Variation in the present-day CR element copy number for different subfamilies (supplementary fig. S2, Supplementary Material online) may indicate variable replication frequencies and synonymous substitution rates for different CR subfamilies. However, with evidence that all of the CR subfamilies have existed for the past 50–70 Myr (Wolfe et al. 1989) (since the divergence of the panicoid and oryzoid lineages), and assuming that element removal occurs at a constant rate, necessitating a certain minimal retrotransposition rate for retention of a CR lineage in any given genome, it can be argued that the application of a constant substitution rate over evolutionary time is justifiable at this time.

The divergence time estimates of oryzoid CR4, CR5, and CR6 elements from their respective panicoid counterparts were slightly higher than that of the CR2 pairs (and possibly CR3 clade, for which there were few data points) that match more closely to the presumed divergence time of the oryzoid and panicoid lineages (supplementary fig. S5, Supplementary Material online). This difference could be due to a higher substitution rate for the pericentromeric CR4, CR5, and CR6 clades, although it may simply reflect the fact that gene trees are often older than the species tree (Nichols 2001).

### Evolutionary History of Grass CRs Based on Divergence Times

Grass CRs belong to group A of the chromoviral CRM clade (Neumann et al. 2011). Homologs of all six maize CR subfamilies have been identified in oryzoid and panicoid lineages and their divergence times may help understand their evolutionary history. A model of CR evolution based on the estimated divergence times of extant CR subfamilies is shown in figure 4. The divergence time of CRA3 of *Arabidopsis* to CR subfamilies of grasses (from 163.879 ± 20.921 to 227.856 ± 58.365 Ma) is consistent with the estimated monocot–dicot divergence time of approximately 200 ± 40 Ma (Wolfe et al. 1989). The divergence time of the most distant members within CR3, CR4, CR5, and CR6 lineages ranges from 80.210 ± 8.856 to 97.250 ± 13.580 Ma (supplementary table S6, Supplementary Material online), which supports the existence of these lineages prior to the pan-grass whole-genome duplication approximately 70 Ma (Paterson et al. 2004). In contrast, the divergence time of most the distant members within the CR1 and CR2 lineages is only 46.075 ± 6.658 Ma and 51.135 ± 4.919 Ma, respectively. Nevertheless, the divergence time of the CR1 and CR2 lineages relative to the other CR lineages (i.e., CR3, CR4, CR5, and CR6) is at least approximately 108.027 ± 10.294 and 82.220 ± 5.929 Ma, respectively (supplementary table S7, Supplementary Material online), which indirectly supports the existence of CR1 and CR2 lineages prior to pan grass whole-genome duplication. Thus, the six distinct subfamilies of grass CRs diverged after the monocot–dicot split approximately 200 Ma (Wolfe et al. 1989) but before the pan-grass whole-genome duplication. Furthermore, the average divergence time estimates group the six CR subfamilies into three distinct groups: The CR5/CR6 lineage, the CR1 lineage, and CR4/CR3/CR2/cereba lineage. The CR5/CR6 lineages diverged first (133.952 ± 16.002 to 174.186 ± 16.058 Ma), followed by the divergence of the CR1 lineage (94.908 ± 8.700 to 110.145 ± 10.080 Ma), and the CR4/CR3/CR2 lineage (82.220 ± 5.929 to 102.758 ± 8.253 Ma). The pooid-specific cereba lineage diverged most recently from the CR2 lineage (65.631 ± 4.389 Ma) and represents the CR2 ortholog in the pooid lineage.

### The Role of CR Elements in Grass Centromeres

The close association of some CR subfamilies with active grass and other centromeres, as well as the ability of retrotransposons to carry a substantial amount of noncoding DNA, raises the intriguing question of whether the CR elements in particular have evolved not only to target active centromeres for their integration into the genome but may actually also carry DNA sequences, possibly between species, that are able to seed centromere formation. This would not contradict existing data on plant centromere function.

## Conclusion

Detailed phylogenetic analysis of CR elements in the grasses illustrates that HT and interelement recombination are important factors in the evolution of CRs. Although only a fraction of HT events can be detected and many recombination events do not yield elements with increased fitness, it is becoming clear that both mechanisms contribute significantly to the evolution of CR elements, possibly by enabling retrotransposons to circumvent the otherwise tight proliferation control mechanisms of the host genome. Key questions that remain to be answered include the exact mechanism of HT (virus-like particle, pollen mediated, or other), as well as its contribution to the non-CR retrotransposon repertoire and genome evolution in plants. The repeat-rich high-quality genome sequences that are being generated now will help answer these questions.

## Supplementary Material

Supplementary files S1 and S2, tables S1–S7, and figures S1–S5 are available at *Genome Biology and Evolution* online (http://www.gbe.oxfordjournals.org/).
